# Influence of Starch-Granule Diameter on Lasing Thresholds of the Molecular Rotor Thioflavin T in a Dual-Cavity Fabry–Perot/Whispering Gallery Mode

**DOI:** 10.1021/acsomega.5c04338

**Published:** 2025-08-20

**Authors:** Katarzyna Szajko, Konstantin Rusakov, Piotr Hanczyc

**Affiliations:** 1 Plant Breeding and Acclimatization Institute - National Research Institute in Radzików, Młochów Division, Platanowa 19, Młochów 05-831, Poland; 2 Faculty of Construction and Environmental Engineering, 49561 Warsaw University of Life Sciences, Warsaw 02-776, Poland; 3 Center of Cellular Immunotherapies, 49561 Warsaw University of Life Sciences, Warsaw 02-786, Poland; 4 Institute of Experimental Physics, Faculty of Physics, 49605 University of Warsaw, Pasteura 5, Warsaw 02-093, Poland

## Abstract

A dual-cavity lasing platform is reported in which thioflavin T (ThT), a rotor-sensitive molecular probe, is employed to map molecular-crowding effects within starch granules via coupled Fabry–Perot (FP) and whispering gallery mode (WGM) resonances. In this architecture, global standing-wave feedback is furnished by a planar FP cavity, while size-tunable WGMs are supported by ThT-coated starch granules. Granules were sorted into five diameter classes (<20, 20–30, 30–40, 40–60, and >60 μm), and lasing thresholds alongside fluorescence lifetimes were determined. Lasing was observed only when a WGM eigenfrequency overlapped spectrally with an FP longitudinal mode, rendering the threshold exquisitely sensitive to granule size, local viscosity, and chromophore packing. The lowest lasing threshold (approximately 11 μJ) was exhibited by the 20–30 μm granules, a “sweet-spot” attributed to optimal optical confinement and maximal steric hindrance of ThT rotation. Increased radiative losses were encountered in smaller granules, whereas larger particles (>60 μm) afforded excess rotational freedom in ThT that inhibited stimulated emission. Time-resolved fluorescence measurements corroborated these trends, with lifetimes reaching τ_avg_ = 0.57 ns in the optimal size regime. Granule diameter is thus established as a precise knob for tuning lasing behavior in crowded systems, with implications for the development of starch-WGM-based photonic sensing in biomolecular processes.

## Graphical abstract

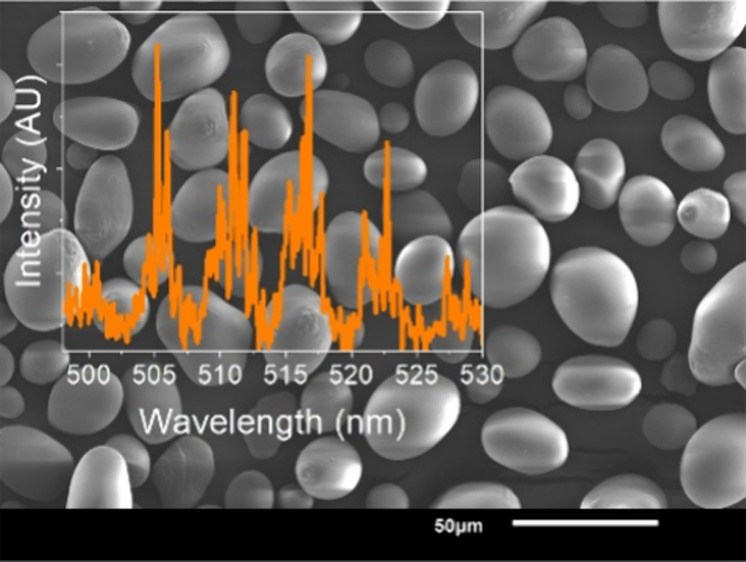

## 1 Intoduction

Starch is a polysaccharide composed primarily of amylose and amylopectin.^1^ It is one of the most abundant and renewable biopolymers found in nature.^2^ It serves as the primary carbohydrate reservoir in many plants and is stored in the form of discrete, semicrystalline granules of varying morphology,^3^ size,^4^ and composition.^5^ Owing to its renewable origin, biodegradability, and relatively low cost, starch and its derivatives have attracted considerable attention for diverse industrial applications ranging from the food sector to pharmaceutical formulation, and more recently, nanotechnology.^6^ Within the realm of nanotechnology, starch granules can act as natural nanoscale frameworks due to their hierarchical structure, tunable surface chemistry, and ability to encapsulate and release molecules in a controlled fashion.^7^

Despite these advantageous features, the use of starch-based systems as nanomaterials requires an in-depth understanding of the molecular-scale interactions^8,9^ that govern their functionality.^10,11^ One critical yet sometimes overlooked factor in these systems is molecular crowding.^12^ Molecular crowding refers to the effect of high concentrations of macromolecules in a confined environment, leading to altered diffusion coefficients, reaction rates, and macromolecular conformations compared to dilute conditions.^13^

An excellent tool for probing the effects of molecular crowding and changes in microenvironment within these systems is thioflavin T (ThT).^14–16^ Traditionally known for its application as a fluorescent reporter in amyloid fibril studies,^17^ or more recently DNA^18^ and mucins,^19^ ThT also functions as a molecular rotor whose fluorescence intensity is highly sensitive to the local environment and viscosity.^20^ Specifically, ThT exhibits enhanced fluorescence when rotational motion of its aromatic rings is restricted, making it an ideal probe for detecting viscosity variations or macromolecular confinement.^21^

In this article, we expand our previous research on lasing properties of ThT in Fabry–Perot (FP) cavity^22^ to a hybrid resonator system whereby FP mirror cavity lasing provides free-space standing wave (global feedback) and whispering gallery modes (WGMs) provide local, size-dependent feedback from starch granules decorated with dye molecules.^23^

In the FP cavity, the lasing effect in ThT arises when an active medium provides sufficient optical gain to overcome inherent losses within a resonant cavity,^24^ leading to the generation of coherent and monochromatic green light through stimulated emission^25^ (Figures 1 and 2a). Under these conditions, photons emitted by excited species within the gain medium induce further emission events that are in phase and of the same wavelength, creating a rapid amplification of the light intensity once a pump energy is surpassing the specific threshold level^26^ (Figure 2 b, right inset showing lasing threshold). Once lasing commences, the output light exhibits distinct spectral narrowing and directional emission, both of which are highly sensitive to changes in the local environment^18^ (Figure 2b).

**Figure 1:**
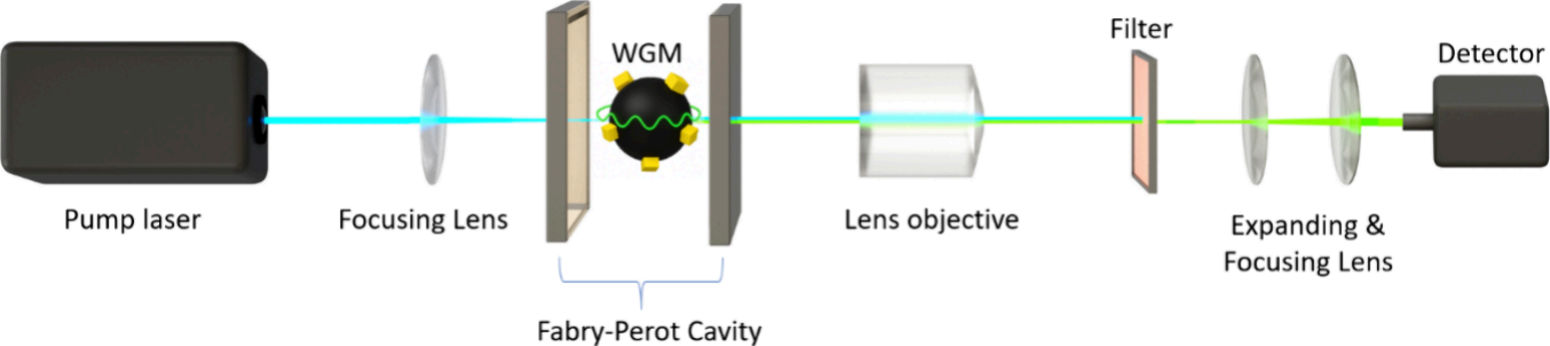
Setup for measuring lasing in Fabry–Perot (FP) cavity with starch decorated with thioflavin T (ThT) dye molecules acting as a whispering gallery mode (WGM) resonator. The lasing signal from the gain medium in the cavities was collected parallel to the excitation beam. A filter was used to block excitation wavelengths >470 nm, allowing only the lasing signal to reach the detector. The lasing signal was collected using a ProEM Excelon camera from Teledyne Princeton Instruments.

**Figure 2:**
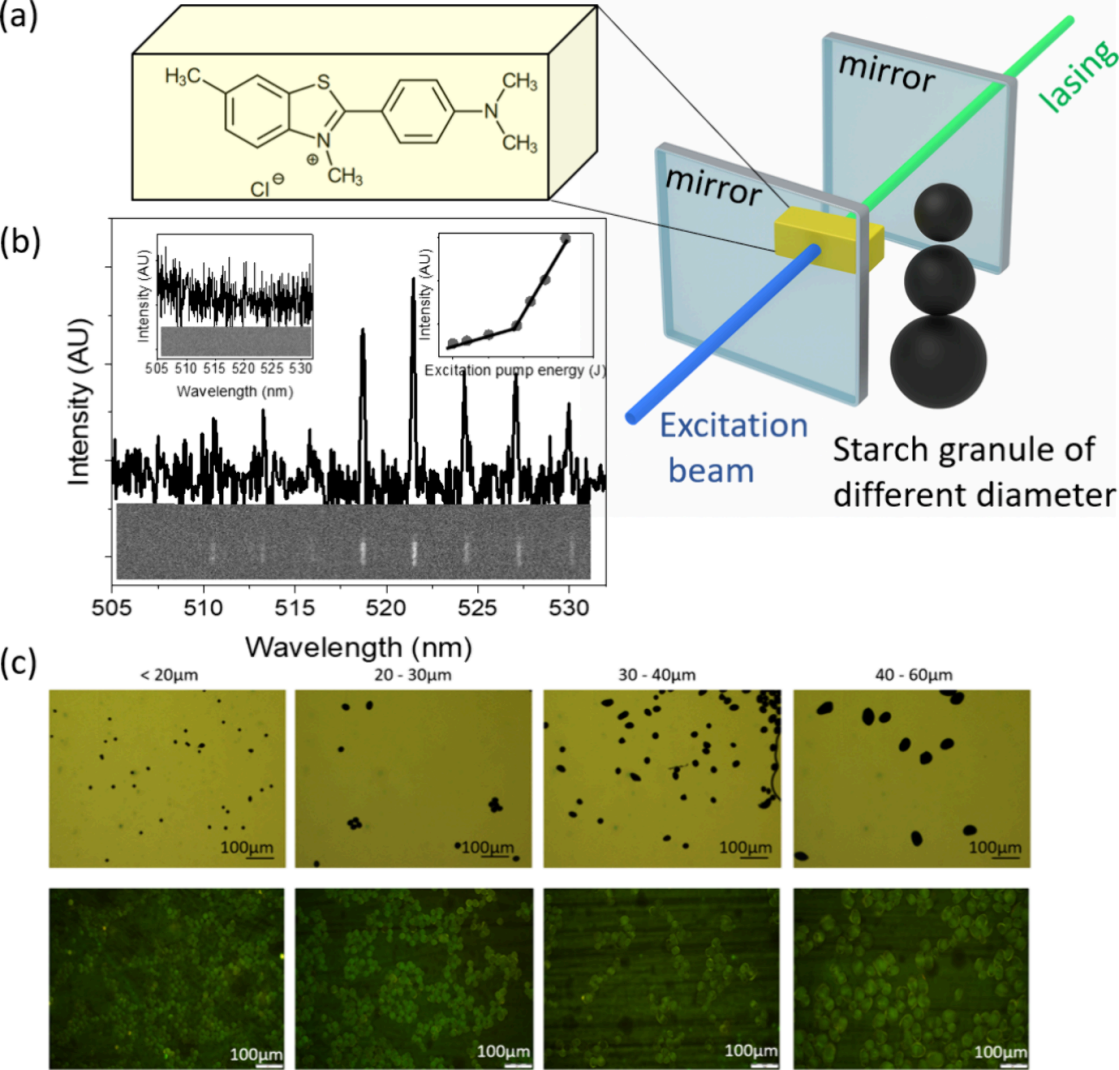
(a) Thioflavin T (ThT) chemical structure in the yellow box reflecting the dye’s appearance in aqueous solution, alongside a schematic of the laser resonator setup comprising two mirrors, starch grains, and ThT molecules. (b) Representative lasing signal (bottom panel) and emission spectrum with multiple peaks emerging above the lasing threshold. The left inset depicts the background signal below the threshold, while the right inset shows an example of the corresponding lasing threshold diagram (λ_ex_ = 420 nm). (c) Optical microscopy images of starch grains illustrating the variation in granules size suspended in 0.1% of Lugol’s solution (top panel), and fluorescence microscopy images of starch grains with different size stained with thioflavin T (botton panel).

In the WGM microresonator, coherent light is generated when the circulating optical field, confined by total internal reflection along the curved starch–water interface, accumulates enough round-trip gain to offset intrinsic scattering and absorption losses.^27^ Photons emitted by ThT molecules that reside within the evanescent field stimulate additional, phase-matched emission as they orbit the granule perimeter, producing a sharp buildup of intensity once the pump fluence exceeds the size-dependent WGM threshold (Figures 1 and 2). After lasing onset, the spectrum collapses into a series of discrete, equally spaced WGM lines whose free-spectral range scales inversely with the granule radius and the output emerges in a characteristic doughnut-shaped pattern normal to the resonator equator.^28^ It is usually described by the equation Δλ = λ^2^/2π*nR*), whereby Δλ is a free-spectral range, the wavelength spacing between neighboring azimuthal WGMs of the same radial order, λ is the central lasing wavelength around which the modes are evaluated, *n* is a refractive index of the starch material, *R* is the radius of the spherical starch resonator (in this study, the starch-granule radius), and 2π is the geometric factor representing the full circumference over which the WGM circulates). Because both the resonance condition and the cavity quality factor (*Q*) depend exquisitely on refractive-index contrast, surface roughness, and local viscosity,^29^ WGM lasing acts as an ultrasensitive reporter of nanoscale alterations in dye packing, interfacial water, and macromolecular crowding within the starch matrix.

The objective of this study is to elucidate how a dual-cavity architecture, combining a planar FP cavity with starch-granule WGMs, modulates the lasing threshold and emission characteristics under macromolecular-crowding conditions.

In this configuration, the FP cavity amplifies minute variations in net optical gain or loss, while WGMs provide a quantitative link between granule size and crowding-induced changes in the effective refractive index, chromophore packing, and interfacial water structure. Coherent emission occurs only when a longitudinal FP mode coincides spectrally with a WGM resonance. Therefore, the lasing threshold and emission line shape serve as ultrasensitive reporters of nanoscale perturbations that are inaccessible to conventional steady-state fluorimetry.

Beyond establishing fundamental relationships among crowding, granule morphology, and optical gain, our findings highlight starch as a promising biomaterial for advanced optoelectronic applications, where shifts in grain size, viscosity, or molecular packing can be harnessed to tune output intensity and wavelength.

## 2 Materials and Methods

### 2.1 Plant Material

The potato cultivar Tajfun originates from the Pomeranian-Masurian Potato Breeding Company in Strzekęcino, Poland, and has highly starch content tubers.^30^ Winter stored tubers were the source of starch granules.

### 2.2 Starch Granules

The tuber sample (approximately 100 g) was ground in a laboratory blender. Starch granules were rinsed from small pieces of tuber cuts. The ground slurry was screened through muslin cloth, where it was left and washed thoroughly with distilled water. After 1 h, the supernatant was decanted in Imhoff-type sedimentation cone. The settled starch was unleashed from the lower layer on the cone. Then, it was resuspended in distilled water. They were decanted on an Erlenmeyer flask three times in ultrapure water for removing the tuber remnants. Finally, the starch was collected and dried at ambient temperature overnight. The 2 g of dried flour was resuspended on 50% EtOH and sieved through cell strainers for 50 mL tubes. For starch-granule selection, four cell strainers were used. Diameters of sieves started from 60 μm via 40 μm and 30 to 20 μm. First, the biggest ones, above 60 μm, were rinsed from the top of 60 μm cell strainer. Granules below the sieve were sieved by 40 μm, and then 30 μm and the last one 20 μm. For each one, starch was collected in new tubes and centrifuged 2800 rpm for 5 min. Finally, the starch samples were collected and dried on 2 mL tubes at room temperature to total drying (overnight).

### 2.3 Light Microscopy

Observation was determined using an optical microscope (Nikon Eclipse E200). The portion of starch samples was suspended in 0.1% of Lugol’s solution to obtain 2 mg/mL for test. One drop of the suspension was placed on a slide glass and then viewed under a microscope at magnification of ×100.

### 2.4 Thioflavin T

Dye was ″UltraPure grade″ purchased from AnaSpec (USA). A stock solution for lasing was 78.4 mM, whereas for time-resolved fluorescence, the final concentration for experiments was 3.1 mM.

### 2.5 Fluorescence Microscopy

The ThT (0.1 mM) dye solutions were freshly prepared in distilled water. The dye solution was immediately added to the samples with the final concentration of 0.2 μM of ThT. About 10 μL aliquots of the samples prestained with ThT were placed on glass slides and air-dried, and images were taken using a fluorescence microscope (Olympus BX50) with a U-FBN filter (excitation wavelengths 470–490 nm, narrow band). Slides with the starch sample were viewed at magnification of ×100.

### 2.6 Time-Resolved Fluorescence Spectroscopy

Time-resolved fluorescence measurements and femtosecond pulses were generated by frequency doubling in a BBO crystal, using the output of an optical parametric amplifier (Orpheus by Light Conversion) pumped by a femtosecond amplifier (Carbide by Light Conversion) at 420 nm. The pulse repetition rate was set at 2 MHz. Spectroscopic measurements were performed using a HORIBA QuantaMaster 8075-11 spectrofluorometer, equipped with a PPD850 photomultiplier (sensitive from 250 to 850 nm) and a DeltaTime kit for time-resolved studies. Emission slits were set to 1 nm, and spectra were corrected for the detector sensitivity. The power of the excitation beam was controlled to prevent saturation and ensure a linear detector response. The instrument response function (IRF) was determined by scattering the excitation beam in a TiO_2_ suspension in water. Fluorescence decay data were analyzed by using Horiba FelixGX software, applying a reconvolution method with the IRF for fitting multiexponential decay models to the experimental data. The average fluorescence lifetime was calculated as the amplitude-weighted mean of the decay times for each component.

### 2.7 Lasing in Fabry–Pérot (FP) Cavities and Whispering Gallery Modes (WGMs)

Lasing spectra were recorded using a femtosecond laser operating at a 0.5 kHz repetition rate, with pulse energies of 400 μJ, and the wavelength used for experiments was 420 nm. The mirrors used for the cavities had nearly 100% transmission in the 400–450 nm range, while reflectance between 470 and 570 nm was approximately 95–99%, centered at 520–530 nm.

The gain medium for lasing in Fabry–Pérot (FP) cavity is thioflavin T (ThT) and starch granules of different diameters sandwiched between two mirrors acting as photonic resonators. The mirror cavity provides strong optical feedback, enabling the detection of subtle molecular changes in the ThT–starch complex. Lasing occurs when the excitation energy is gradually increased, achieved by incrementally adjusting the gray filter with a Thorlabs motor system, while simultaneously monitoring the emission spectrum with a detector. The relationship between the pump energy and emission intensity was plotted to determine the lasing thresholds.

At a specific energy, the ThT emission spectrum narrows significantly and a sharp increase in light intensity is observed. The pump energy at which this occurs is defined as the lasing threshold. Typically, when population inversion takes place within the cavity, a narrow lasing peak is observed without background fluorescence. The full width at half-maximum (fwhm) of the lasing spectrum in the cavities is typically only a few nanometers wide.

## 3 Results and Discussion

Thioflavin T (ThT) combines pronounced sensitivity to microviscosity with its inherent capability for light amplification in Fabry–Perot (FP) mirror cavity in a molecular crowding environment. Here, we expand the system by creating a dual-cavity architecture whereby a light feedback from the FP cavity is supported with whispering gallery modes (WGM) governed by starch granules grouped by size: smaller than (i) <20 μm, (ii) 20–30 μm, (iii) 30–40 μm, and (iv) 40–60 μm and (v) above 60 μm (Figure 2). By exploiting ThT’s rotor-like properties, it is therefore possible to obtain insights into lasing effects in the crowded nanostructured starch systems, facilitating the rational design and optimization of starch-based functional materials.

Figure 3 presents charts of lasing threshold measurements for ThT added into starch granules of progressively increasing diameters. Above 60 μm, no lasing was detected (threshold levels summarized in Table 1). Because all size classes were evaluated using the same Fabry–Perot cavity, the global mirror feedback is effectively constant, and the observed variations in threshold arise solely from the size-dependent WGM feedback. Granule diameter thus controls the local WGM resonance conditions, which in turn dictate ThT molecular packing density and microviscosity and therefore the lasing efficiency. Notably, granules in the 20–30 μm range exhibited the lowest lasing threshold, indicating that this size regime provides the optimal balance of steric confinement and local viscosity required to suppress ThT’s intramolecular rotation and maximize stimulated emission.

**Figure 3:**
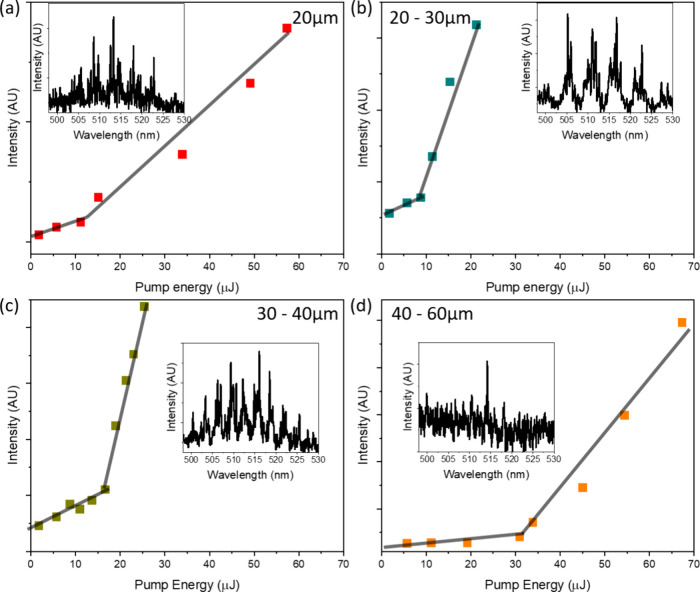
Charts of lasing thresholds of ThT in the presence of (a) <20 μm, (b) 20–30 μm, (c) 30–40 μm, and (d) 40–60 μm, λ_ex_ = 420 nm. For each size class, the threshold was determined from three consecutive experiments.

**Table 1:** Starch Granule Size and Lasing Thresholds of Thioflavin T *C*_ThT_ = 26 mM Dissolved in Water, λ_ex_ = 420 nm

starch grain size (μm)	lasing thresholds (μJ)
<20	15 ± 1.2
20–30	11 ± 0.7
30–40	19 ± 2.1
40–60	31 ± 2.6
>60	

Since the lasing threshold results show that granule diameter is the critical parameter controlling the WGM landscape and lasing efficiency of ThT in the dual-cavity system, it is important to delve deeper into understanding the molecular organization in the condensed phase. Smaller granules, particularly those below 20 μm in diameter, pack more densely within the fixed FP mode volume, thereby intensifying molecular crowding and enhancing the WGM feedback. However, excessive crowding can perturb energy-transfer pathways crucial for establishing population inversion and simultaneously promote nonradiative decay channels, ultimately increasing lasing thresholds. Conversely, granules larger than 40 μm exhibit reduced local crowding, leading to a decreased steric confinement of ThT molecules. This reduction in steric hindrance allows greater rotational freedom of ThT, diminishing the conformational locking essential for a high fluorescence quantum yield and efficient stimulated emission. Consequently, larger granules require significantly higher pump fluences to achieve lasing or, in the case of granules exceeding 60 μm, fail entirely under identical excitation conditions. The optimal performance was thus observed for starch granules in the intermediate size range of 20–30 μm, a regime recognized as a ″sweet-spot″ for WGM microresonators, where mode volume, optical confinement, and *Q*-factors (typically 10^3^–10^4^) synergistically overlap with the pump focus.^31,32^ In this size range, the circulating optical field achieves sufficient intensity to facilitate population inversion while remaining confined to a shell thin enough to impose strong steric restrictions on ThT molecules. This balanced interplay among optical confinement, mode overlap, molecular crowding, and steric hindrance yields the lowest lasing thresholds and most efficient stimulated emission within the studied dual-cavity configuration.

As a control experiment for lasing results, time-resolved fluorescence measurements provided additional insight into the correlation between ThT local environment and lasing (Figure 4). In the presence of starch grains of size 40–60 μm, as well as those exceeding 60 μm, ThT exhibited lifetimes similar to those of the free dye in aqueous solution, suggesting that its molecules remain largely unhindered and free to rotate. This unrestricted molecular motion aligns with the high lasing thresholds or complete lack of lasing observed for these larger grains, reinforcing the notion that crowding-induced restriction of ThT intramolecular rotation is crucial for facilitating stimulated emission.

**Figure 4:**
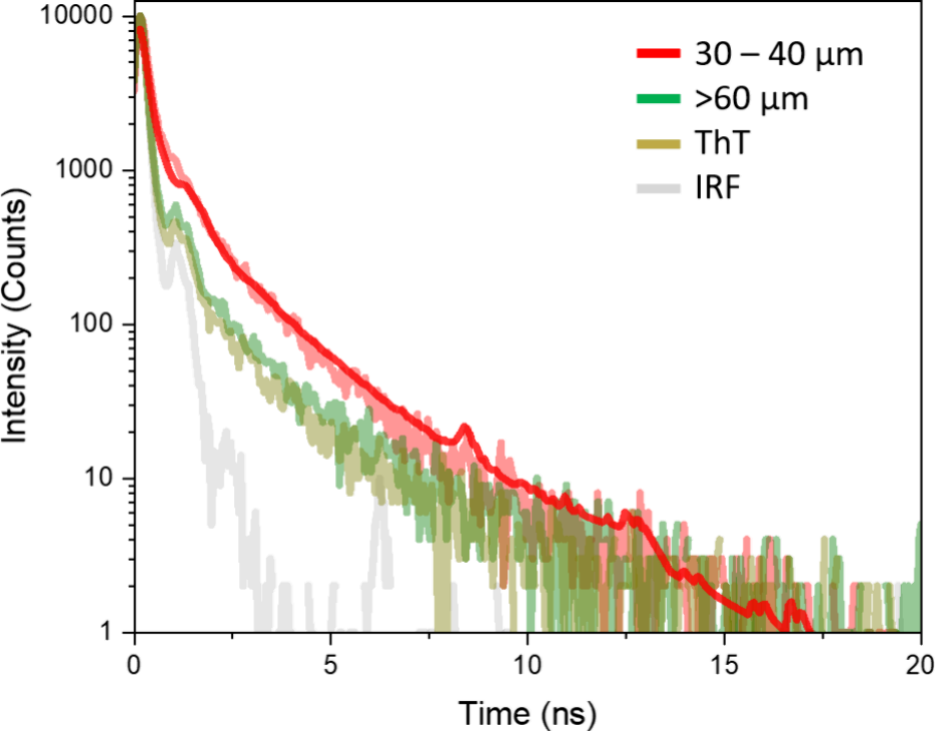
Example of the fluorescence decay of ThT with small starch grains 30–40 μm μm (red decay with red fitting curve) and large grains >60 μm (green decay). For comparison, decay of ThT dissolved in water (yellow) and the instrument response function (IRF, at 420 nm (gray), samples were excited λ_ex_ = 420 nm and lifetimes were collected at λ_em_ = 495 nm.

Conversely, smaller granules (<20, 20–30, and 30–40 μm) required a two-component fit to describe their fluorescence decay, yielding average lifetimes of 0.63, 0.57, and 0.64 ns, respectively (Table 2). These values imply a partially hindered rotational environment, consistent with enhanced microviscosity in the presence of smaller starch granules. Notably, the grain size of 20–30 μm, which yielded the lowest lasing threshold, also demonstrated slightly shorter lifetime than the >20 and 30–40 μm, highlighting the link between moderate levels of crowding, optimal restriction of ThT rotation, and effective light amplification.

**Table 2:** Fluorescence Decay Times (τ_i_) of ThT-Stained Starch Grains Obtained by Fitting Two-Exponential Functions to Fluorescence Decays, λ_ex_ = 420 nm, Instrument Response Function (IRF)

grain size (μm)	τ_1_ (ns)	τ_2_ (ns)	τ_avg_ (ns)
ThT in water^a^	>IRF	>IRF	
<20	0.18 ± 0.01 (0.68)	1.6 ± 0.01 (0.32)	0.63
20–30	0.13 ± 0.01 (0.72)	1.7 ± 0.01 (0.28)	0.57
30–40	0.18 ± 0.01 (0.66)	1.5 ± 0.01 (0.34)	0.64
40–60^a^	>IRF	>IRF	
>60^a^	>IRF	>IRF	

a Signal below the IRF.

## 4 Conclusions

In this work, we have demonstrated that the lasing behavior of thioflavin T (ThT) in starch granules can be precisely tuned by exploiting a hybrid Fabry–Perot/whispering gallery mode (FP/WGM) resonator architecture. By sorting granules into five diameter classes (<20, 20–30, 30–40, 40–60, and >60 μm) and embedding them within an FP cavity, we showed that coherent emission occurs only when a size-dependent WGM eigenfrequency overlaps a longitudinal FP mode. This nested-cavity design renders the lasing threshold exquisitely sensitive to granule diameter, revealing the optimal “sweet-spot” for lasing at 20–30 μm. In this regime, strong optical confinement combines with maximal molecular crowding to suppress ThT intramolecular rotation and drive efficient stimulated emission at minimal pump fluence.

Our systematic threshold measurements further elucidate the competing physical effects at play: Granules smaller than 20 μm suffer excessive radiative leakage and limited gain volume, while those larger than 40 μm exhibit reduced steric hindrance that weaken lasing. The result is a clear, reproducible dependence of threshold on granule size, which can serve as a quantitative optical assay of microviscosity, refractive-index contrast, and dye-packing density within nanostructured starch systems.

Beyond fundamental insights into crowding-mediated photophysics, our findings open avenues for the rational design of starch-based photonic materials and sensors. Future work will focus on extending this approach to apply ThT and starch in studies of biomolecular systems such as protein aggregation, taking advantage of starch acting as WGMs. The dual-cavity FP and starch WGM platform thus provides a versatile, high-resolution tool for probing and engineering the interplay of structure, chemistry, and optics in soft, crowded media.
